# Liberation from Dialysis Dependence in a Patient with HIV-Associated Nephropathy (HIVAN) after Combined Antiretroviral Therapy (cART)

**DOI:** 10.1155/2020/7294765

**Published:** 2020-03-15

**Authors:** Virin Ramoutar, Raafat Makary, Malleswari Ravi, Leighton James, Charles Heilig

**Affiliations:** ^1^Nephrologist & Infectious Disease Specialist, Cumberland Kidney Specialists, Cookeville, TN, USA; ^2^Pathology Department, University of Florida, Jacksonville, FL, USA; ^3^Division of Infectious Disease, University of Florida, Jacksonville, FL, USA; ^4^Division of Nephrology, Medical College of Georgia, Augusta University, Augusta, GA, USA; ^5^Division of Nephrology, University of Florida, Jacksonville, FL, USA

## Abstract

Prior to the advent of combined antiretroviral therapy (cART), human immunodeficiency virus-associated nephropathy (HIVAN) was inevitably associated with rapidly progressive renal failure and dialysis dependence. HIV-1 seropositive patients often met with untimely deaths due to complications of end-stage renal disease (ESRD), opportunistic infections, or other HIV-related end-organ failure. Although the association between cART and improved outcomes in HIVAN has been recognized for over 20 years, no randomized trials have specifically examined this effect to date. In terms of reversal of dialysis-dependent renal failure after cART initiation, only a handful of case reports exist. The authors report a case of a 44-year-old Latino male requiring thrice-weekly haemodialysis in the setting of biopsy-proven HIVAN who was able to stop dialysis in 7 months after being initiated on cART.

## 1. Introduction

HIVAN, the classic renal pathology associated with HIV infection, was first described in 1984 as a sequela of acquired immune deficiency syndrome (AIDS) [1, 2]. It is the most common cause of ESRD in HIV-1 seropositive patients [3] and manifests histologically as a collapsing form of focal segmental glomerulosclerosis (FSGS) accompanied by microcystic tubular dilatation and interstitial inflammation [4]. The prevalence ranges from 3.5% in clinical studies to 12% on postmortem evaluation [3], and it occurs more frequently in patients of African descent [5, 6]. Risk factors include CD4 cell count of less than 200 cells/mm^3^ and a high viral load, but HIVAN has been seen in acute HIV infection [7, 8] as well as with undetectable viral loads [9]. The benefit of cART in HIVAN is based on observational data with isolated cases demonstrating remission within a few weeks [10, 11]. In terms of dialysis-dependent HIVAN, a literature search yielded only 3 prior published cases in which cART initiation improved renal function enough to reverse dependence on renal replacement therapy [12–14]. Two of these 3 cases were African-American males with no ethnicity reported by Winston et al. [13]. In this report, a 44-year-old Latino male with biopsy-proven HIVAN experienced reversal of dialysis dependence 7 months after cART initiation without the use of corticosteroids.

## 2. Case Presentation

A 44-year-old Latino male presented to the ER with complaints of generalized malaise, low grade fever, and watery stools for 1 to 2 weeks. He passed bowel movements 5 times per day without frank blood, melena, or mucus. He had a past medical history of chronic hypertension (HTN) and chronic kidney disease (CKD) with a baseline serum creatinine (sCr) in range 2.0 to 2.5 mg/dL. On presentation, his serum bicarbonate level was 9 mmol/L with blood urea nitrogen (BUN) 125 mg/dL and sCr 13.8 mg/dL. He had been initiated on lisinopril for HTN and to mitigate CKD progression 1 month prior to onset of symptoms. This was discontinued on presentation to the hospital and was his only prescribed medication. He denied any history of nonsteroidal anti-inflammatory drug (NSAID) use or intravenous drug abuse. Complete blood count revealed hemoglobin 7 g/dL, white cell count 4,800/mm^3^, and mild thrombocytopenia 78,000/mm^3^ (no abnormalities seen on peripheral smear). Urine microscopy revealed 2 normomorphic red blood cells, 9 white cells, and 3 hyaline casts per high power field. The spot urine protein to creatinine ratio was 10,417 mg/g, and urinalysis tested positive for protein at 2,000 mg/dL. He was volume expanded with an isotonic solution of sodium bicarbonate (150 mEq) in a litre of 5% dextrose solution. His serum bicarbonate normalized, but BUN remained elevated at 106 mg/dL with sCr 13.49 mg/dL. Urine culture was positive for pan-susceptible *Enterococcus faecalis*. He received a 10-day course of amoxicillin to sterilize the genitourinary tract in anticipation of a renal biopsy.

A renal ultrasound with Doppler sonography was performed to exclude obstructive uropathy and renal artery stenosis. There was no evidence of either condition, and both kidneys demonstrated increased echogenicity. The left kidney was measured at 10.3 cm and the right at 9.9 cm in the longitudinal axis. Serum complements were within normal range. Laboratory investigations returned negative for syphilis, hepatitis B and C, rheumatoid factor, antinuclear antibody (ANA), antineutrophil cytoplasmic antibody (ANCA), and monoclonal paraproteins in both urine and serum. Antistreptolysin O titres were within normal limits. HIV-1 Western blot returned positive with a viral load of 664,000 copies/ml and CD4 count of 40 cells/*μ*L. Blood and stool samples did not yield any other infectious aetiology. His diarrhoea resolved within 36 hrs of hospital admission, but he exhibited continued anorexia and intermittent nausea in the setting of persistent azotaemia and oliguria (<800 cc urine/day). Haemodialysis was initiated via a tunnelled cuffed catheter.

**Figure 1:**
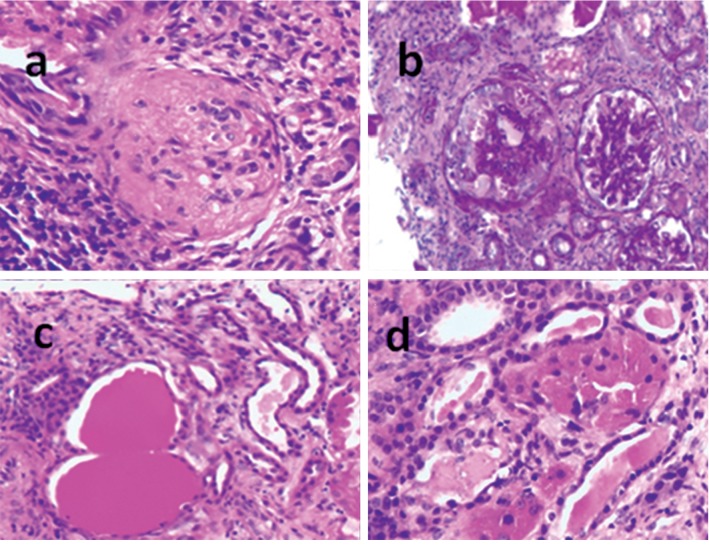
Haematoxylin and eosin stain (×20 magnification) showing (a) global to near global glomerulosclerosis, (b) collapsed glomerular tuft, (c) tubular microcystic dilatation, and (d) distended tubular epithelium from protein resorption droplets.

**Figure 2:**
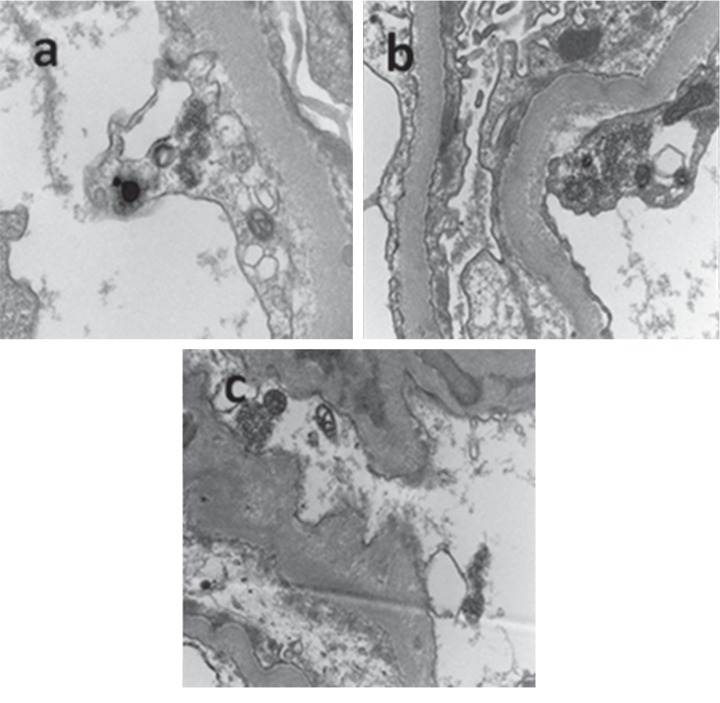
Electron microscopy (×35200–56200 magnification) showing (a) tubuloreticular inclusions (TRIs) in the endothelium of the glomerular capillaries, (b) thickened glomerular basement membrane with foot process effacement, and (c) absence of amorphous immune-type deposits (electron dense deposits).

A percutaneous renal biopsy was performed in light of proteinuria and HIV seropositivity. The biopsy revealed collapsing glomerulosclerosis, tubular dilatation (Figure 1), tubuloreticular inclusions, thickened glomerular basement membrane, foot process effacement, and absence of electron dense deposits (Figure 2). 20 glomeruli were sampled, and 5 (25%) were globally sclerotic. A variable degree of focal segmental glomerulosclerosis (FSGS) with mesangial hypercellularity was present in the remaining glomeruli with collapsing features noted in 6 (30%) glomeruli. Immunofluorescence was negative, and there was no eosinophilic infiltrate. Additional findings included mild-to-moderate arteriosclerosis with moderate chronic interstitial inflammation comprising lymphocytes and plasma cells.

**Figure 3:**
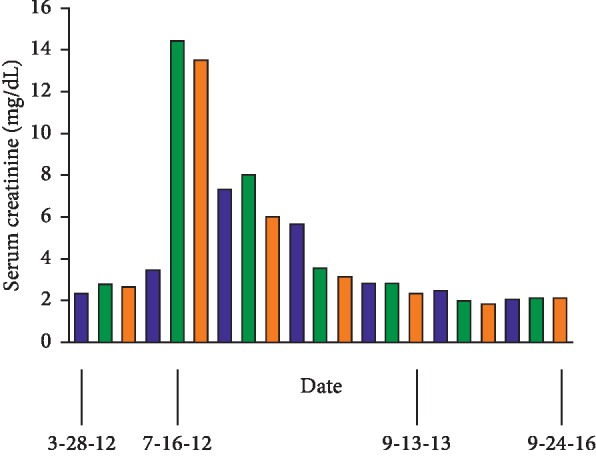
Demonstration of the change in serum creatinine over time showing return to stable baseline CKD 4 after cART initiation with sCr of approximately 2 mg/dL.

Human leukocyte antigen (HLA) B5701 testing returned negative, and the patient was initiated on cART with abacavir, lamivudine, atazanavir, and ritonavir prior to discharge. The patient was discharged with outpatient haemodialysis thrice weekly and followed up with infectious disease for HIV treatment. In 3 weeks, his viral load decreased to 17,000 copies/ml and then to 150 copies/ml in 2 months. Complete virologic suppression (undetectable viral load) was documented 11 months after cART initiation. His CD4 count improved to >200 cells/*μ*L in 2 months. After 7 months on haemodialysis, it was noted that the patient was no longer oliguric and had no significant interdialytic change in BUN and sCr. Haemodialysis was discontinued, and the patient remained in stage 4 CKD range (as demonstrated in Figure 3).

## 3. Discussion

Classic HIVAN is identified by the following pathognomonic constellation of features: collapse of glomerular capillaries, visceral glomerular epitheliosis, podocyte hypertrophy and proliferation, mesangial prominence and hypercellularity, endothelial tubuloreticular inclusions (TRIs), and microcystic tubules [2,15]. It remains the most common renal histology finding in the HIV-infected [16], despite a decline in prevalence owing to the widespread use of cART. According to the US Renal Data System (USRDS), there has been 60% reduction in the risk of ESRD associated with HIVAN following the introduction of cART [17].

Three major groups of nephropathy are seen in HIV-positive patients: classic HIVAN, HIV-associated thrombotic microangiopathy, and HIV-associated immune-mediated glomerulonephritides [18]. The clinical presentation and pathological findings in this case were consistent with classic HIVAN. The duration of HIV infection for the patient was unknown, and although traditionally thought to occur later in the disease course, renal involvement may be seen as early as during acute seroconversion [13]. The exact pathogenesis of HIVAN and the mechanism by which cART improves renal function are unknown. Murine studies suggest that transgene expression is responsible for the histopathologic changes seen in HIVAN, and this process is mitigated by cART initiation [13, 19].

HIVAN is viewed as a disease that occurs as a direct effect of renal cellular HIV-1 infection and more so of HIV-1 gene products on the kidney. The effect of the HIV viral load in the circulation on this process remains unclear. Reversal of dialysis dependence occurred in this case 4 months prior to complete virologic suppression. In the case by Vasquez et al., complete virologic suppression did not occur, not even 16 months after cessation of haemodialysis [14]. Wali et al. noted that after 13 weeks of cART and 12 weeks of haemodialysis, reversal of dialysis-dependence occurred with a detectable viral load (<500 copies/ml) [12]. Winston et al. noted that complete virologic suppression occurred within 6 weeks of cART, and in this case, haemodialysis was only required for 2-3 weeks [13].

In this case, only 5 (25%) of the sampled glomeruli were sclerotic without notable interstitial fibrosis or tubular atrophy (IFTA). Prior reports noted “patchy interstitial fibrosis” [13] and “mild periglomerular fibrosis” [12], while Vasquez et al. noted the presence of glomerular sclerosis but did not qualify its extent [14] (images of slides in this paper did not show significant IFTA). The initiation of cART before the onset of significant fibrosis appears to play a major role in recovery from and possibly progression to, dialysis dependence. However, there remains a paucity of data on this subject and despite use of cART many HIV patients progress to ESRD. Even in this subset, the survival of HIV-infected haemodialysis patients on cART is superior to those on suboptimal antiretroviral regimens or no treatment at all [20, 21].

In terms of treatment, ACE inhibitors and angiotensin II receptor blockade is recommended to slow disease progression in patients who have not shown significant improvement with cART alone. Renin-angiotensin blockade may help decrease proteinuria and slow progression of fibrosis, and this has been previously demonstrated in transgenic mice [22]. Glucocorticoids may provide benefit to cases with histologic evidence of dense tubulointerstitial inflammatory infiltrates. However, the evidence for this is limited, and their role remains controversial [23, 24]. Glucocorticoid therapy was neither employed in this case, nor the three prior referenced cases of HIVAN that recovered from dialysis dependence.

The Infectious Diseases Society of America (IDSA) recommends cART for all HIV-infected at time of diagnosis and referral to nephrology when there is albuminuria >300 mg per day, a clinically significant decline in glomerular filtration rate (GFR) or haematuria of renal origin [24]. Renal biopsy is encouraged as a definitive diagnosis may inform prognosis and treatment options. Further, the specificity and sensitivity of noninvasive testing is limited. The existing data have solidified the role of cART in the HIV-infected, regardless of whether renal disease is present or not. However, the interplay of host genetic factors and viral transgene expression that results in variable progression of nephropathy in those diagnosed with HIVAN remains largely unexplored.

## 4. Conclusion

The onset and progression of HIVAN remains variable, despite the widespread use of cART and an overall improved prognosis for the HIV-infected population. The authors describe the 4^th^ case, as indexed in PubMed, of a patient with HIVAN who was liberated from dialysis dependence after the initiation of cART. There is a paucity of data on the subject and a lack of research into host genetic factors to account for the variable course of HIVAN in the era of cART. Further research is encouraged in this area since early cART initiation, prior to the onset of significant fibrosis in HIVAN, may prevent or reverse progression to ESRD.
